# Supplementary material for: Lower limb chronic edema management program: Perspectives of disengaged patients on challenges, enablers and barriers to program attendance and adherence
Source: PLoS One. 2019 Nov 25;14(11):e0219875. doi: 10.1371/journal.pone.0219875 (PMC6876834; doi:10.1371/journal.pone.0219875)
Supplement: S1 File — (PDF) [file pone.0219875.s001.pdf]

Lower limb chronic edema management program: Perspectives of disengaged patients on challenges, enablers and barriers to program attendance and adherence.

Interview preparation and interview question guide

Preparation

Team→ trial with patients → health professionals from outside of area

Aims of research and interview

Objective 1: To establish their baseline current health-related quality of life (Self-administered LYMQOL- Lower limb edema Questionnaire).

Objective 2: To establish their baseline health-related resilience status (Self-administered Brief Resilience Scale Questionnaire).

Objective 3: To explore patients' perspectives of their experience in management of their chronic oedema.

Objective 4: To explore the challenges, enablers and barriers faced in management of their chronic oedema.

Objective 5: To explore their perception of progress in their chronic oedema healthcare needs and perception of their role in management of their condition.

Checklist

□ Ensure participant has completed 2 questionnaires and sociodemographic survey.

Please clarify the following at the start of the interview:

□ Marital status

□ Living status- alone, live with spouse, live with friend?

□ How long have you had leg swelling (lymphoedema)?

□ Who diagnosed this condition?

□ How long did you have leg swelling before you were referred to this clinic?

□ How many bouts of infection in the past year?

□ Any hospital admissions from infection in the past year?

Interview question guide

Experience with the program (Exploratory questions) – Objective 3 and 4

What treatment were you offered for your leg swelling?

How helpful has the treatment been?

If yes, in what way?

If no, how do you think we can do better?

Did the treatment and care meet your expectations? Why or why not?

What aspects did you like about the care you received?

What aspects did you like the least?

What did you find most useful? What did you find least useful? Why?

Were you able to continue any of the treatment at home?

If yes, please give examples

If no, please indicate why you were unable to continue the treatment at home

If no, what was stopping you? Rank them in order of difficulty?

Are there any problems in following the care and advice about your leg swelling?

Please tell me what they are?

How do you feel about your leg at this moment? Has this changed since you started the program? If so, in what ways?

Has the clinic and treatment met your expectations?

Perception of contextual or personal factors (Exploratory questions) -Objective 5

How do you tell you are making progress with your leg swelling?

How would you personally measure progress with your treatment?

What would you consider to be a good meaningful outcome? A success?

What do you think you can do to reach this outcome?

What are the challenges you face?

To what extent do you feel in control of your condition?

over your treatment?

over the progression of your leg swelling?

How different are your goals from the goals of your therapists?

What else would you like to share regarding your care and the outpatient clinic?
