# Supplementary material for: Lower limb chronic edema management program: Perspectives of disengaged patients on challenges, enablers and barriers to program attendance and adherence
Source: PLoS One. 2019 Nov 25;14(11):e0219875. doi: 10.1371/journal.pone.0219875 (PMC6876834; doi:10.1371/journal.pone.0219875)
Supplement: S2 File — (PDF) [file pone.0219875.s002.pdf]

Lower limb chronic edema management program: Perspectives of disengaged patients on challenges, enablers and barriers to program attendance and adherence.

NVivo higher level nodes and sub-nodes

Name	Files	References	Created On	Created By	Modified On	Modified By
Effectiveness	6	19	25/10/2018 12:26 PM	AB	8/03/2019 3:37 PM	AB
Reduce swelling	1	1	6/12/2018 5:09 PM	AB	6/12/2018 5:09 PM	AB
Better movement	1	1	6/12/2018 5:10 PM	AB	6/12/2018 5:11 PM	AB
Ideal or ideal treatment	6	12	28/10/2018 5:20 PM	AB	8/03/2019 3:42 PM	AB
Expectations of therapy	6	19	28/10/2018 5:40 PM	AB	8/03/2019 3:53 PM	AB
Barriers to care and self (at home) care	6	21	25/10/2018 12:27 PM	AB	8/03/2019 3:40 PM	AB
Challenges with recommended approach	6	17	28/10/2018 5:25 PM	AB	8/03/2019 3:49 PM	AB
Stigma (self consciousness) LK_emotion (e.g.frustration)	6	36	28/10/2018 5:30 PM	AB	8/03/2019 4:05 PM	AB
Childhood experiences	1	1	15/11/2018 9:45 AM	AB	15/11/2018 9:45 AM	AB
Seating in public spaces	2	4	15/11/2018 9:53 AM	AB	15/11/2018 10:02 AM	AB
Conscious of appearances	2	6	15/11/2018 9:58 AM	AB	15/11/2018 10:01 AM	AB
inappropiate focus on legs-stockings	1	2	15/11/2018 10:17 AM	AB	15/11/2018 10:19 AM	AB
Frustration with repeated explaining condition	1	1	15/11/2018 10:20 AM	AB	15/11/2018 10:20 AM	AB
Maintain independence	4	14	29/10/2018 12:33 PM	AB	8/03/2019 4:15 PM	AB
Social-psychological implications for individual with Oedema	6	33	18/10/2018 10:44 AM	AB	8/03/2019 4:01 PM	AB
Frustration with physical limitations	1	1	15/11/2018 10:39 AM	AB	8/03/2019 4:17 PM	AB
social implication of debilitation	0	0	15/11/2018 10:21 AM	AB	6/12/2018 5:02 PM	AB
Wheelchair and disability	1	2	6/12/2018 4:54 PM	AB	8/03/2019 4:09 PM	AB
Family interaction	1	1	6/12/2018 4:50 PM	AB	8/03/2019 4:06 PM	AB
Tailor made clothing	1	1	6/12/2018 4:47 PM	AB	8/03/2019 4:08 PM	AB
socioability	1	2	15/11/2018 10:16 AM	AB	8/03/2019 4:08 PM	AB
Poor community understanding	1	3	6/12/2018 4:52 PM	AB	8/03/2019 4:08 PM	AB
House bound or country bound	3	10	6/12/2018 4:46 PM	AB	6/12/2018 4:56 PM	AB
Gardening	1	1	6/12/2018 4:53 PM	AB	8/03/2019 4:07 PM	AB
Psychological support	1	2	15/11/2018 10:31 AM	AB	15/11/2018 10:48 AM	AB

Lack of knowledge about how to treat	2	2	18/10/2018 10:03 AM	AB	8/03/2019 3:30 PM	AB
First presentation	4	7	18/10/2018 10:03 AM	AB	4/11/2018 6:00 PM	AB
motivation to be involved in research	4	5	18/10/2018 10:14 AM	AB	4/11/2018 4:59 PM	LK
Pre Self management strategies	3	5	18/10/2018 10:16 AM	AB	4/11/2018 6:08 PM	AB
Products and sources	5	9	18/10/2018 10:30 AM	AB	8/11/2018 9:24 PM	AB
Ongoing management	5	21	18/10/2018 10:37 AM	AB	10/11/2018 9:37 PM	LK
Post Self management strategies	6	19	18/10/2018 10:42 AM	AB	8/11/2018 9:30 PM	AB
Wound dressing	2	4	11/11/2018 6:07 PM	LK	11/11/2018 6:19 PM	LK
Skin Care	1	1	11/11/2018 6:07 PM	LK	11/11/2018 6:07 PM	LK
Monitor_Medication	3	6	11/11/2018 6:14 PM	LK	11/11/2018 6:23 PM	LK
Rest_Exercise	2	3	11/11/2018 6:15 PM	LK	11/11/2018 6:17 PM	LK
Compression Therapy	0	0	11/11/2018 6:19 PM	LK	11/11/2018 6:19 PM	LK
Compression Bandaging	1	2	11/11/2018 6:12 PM	LK	11/11/2018 6:16 PM	LK
Compression Pump	1	4	11/11/2018 6:21 PM	LK	11/11/2018 6:22 PM	LK
Stocking	1	5	11/11/2018 6:06 PM	LK	11/11/2018 6:13 PM	LK
Wraps	1	1	11/11/2018 6:17 PM	LK	11/11/2018 6:18 PM	LK
Falls Prevention Aids	1	1	11/11/2018 6:20 PM	LK	11/11/2018 6:21 PM	LK
Co-morbidities	5	23	18/10/2018 10:43 AM	AB	10/11/2018 10:00 PM	LK
Hospitalisation	4	7	25/10/2018 12:20 PM	AB	10/11/2018 9:18 PM	LK
Description of oedema	5	7	25/10/2018 12:21 PM	AB	10/11/2018 9:55 PM	LK
Limitations of stockings AB_ wraps	5	13	25/10/2018 12:23 PM	AB	4/11/2018 6:25 PM	AB
alternative places for care	3	6	28/10/2018 5:13 PM	AB	4/11/2018 4:39 PM	LK
Pain pre and post	6	14	28/10/2018 5:15 PM	AB	15/11/2018 3:27 PM	AB
Other possible treatments	5	7	28/10/2018 5:21 PM	AB	10/11/2018 9:20 PM	LK
Reason for CO	6	6	28/10/2018 5:22 PM	AB	4/11/2018 5:58 PM	AB

Impact on mobility and functionality due to CO	6	33	28/10/2018 5:42 PM	AB	14/03/2019 1:02 PM	LK
Reduced distance	5	7	11/11/2018 8:39 PM	LK	11/11/2018 8:54 PM	LK
Mobility aid e.g. Walker, Wheelchair	3	6	11/11/2018 8:39 PM	LK	11/11/2018 8:54 PM	LK
Access to ACROD Parking	3	4	11/11/2018 8:41 PM	LK	12/11/2018 10:25 PM	AB
Altered Activity-unable to do	4	10	11/11/2018 8:45 PM	LK	11/11/2018 8:56 PM	LK
Fatigue	2	3	11/11/2018 8:46 PM	LK	11/11/2018 8:52 PM	LK
Barriers to using clinic or hospital	4	10	29/10/2018 12:12 PM	AB	8/03/2019 3:22 PM	AB
Hospital_Clinic Accessibility	3	7	1/11/2018 11:06 AM	AB	8/03/2019 3:19 PM	AB
Lack of knowledge who to refer to	2	6	29/10/2018 9:54 PM	LK	8/03/2019 4:13 PM	AB
Lack of knowledge of referral process	2	10	29/10/2018 10:23 PM	LK	8/03/2019 4:13 PM	AB
Adviser and most useful view	5	12	18/10/2018 10:35 AM	AB	8/03/2019 3:46 PM	AB
Impact on family	6	14	28/10/2018 5:46 PM	AB	8/03/2019 4:02 PM	AB
Lack of understanding by others	1	3	30/10/2018 9:00 PM	LK	8/03/2019 4:14 PM	AB
Initial diagnosis	6	20	18/10/2018 9:54 AM	AB	11/11/2018 5:31 PM	LK
Vascular Clinic	2	2	11/11/2018 5:31 PM	LK	11/11/2018 5:36 PM	LK
Dermatology	2	2	11/11/2018 5:33 PM	LK	11/11/2018 5:35 PM	LK
Cardiologist	1	1	11/11/2018 5:34 PM	LK	11/11/2018 5:35 PM	LK
Orthopaedic_Infectious Disease	1	3	11/11/2018 5:36 PM	LK	11/11/2018 6:02 PM	LK
First oedema management_and LK_ YEAR	5	15	18/10/2018 9:56 AM	AB	4/11/2018 3:31 PM	LK
Strategies of clinical care	6	41	18/10/2018 9:57 AM	AB	10/11/2018 10:01 PM	LK
Compression Therapy	0	0	11/11/2018 5:39 PM	LK	8/03/2019 3:34 PM	AB
MLD	3	6	11/11/2018 5:41 PM	LK	11/11/2018 5:52 PM	LK
Advice_Monitor	3	9	11/11/2018 5:45 PM	LK	11/11/2018 6:04 PM	LK
Exercise	1	3	11/11/2018 5:55 PM	LK	11/11/2018 5:57 PM	LK
Surgical	1	2	11/11/2018 5:58 PM	LK	11/11/2018 5:58 PM	LK

Perception of progress of condition	0	0	28/10/2018 5:30 PM	AB	15/11/2018 3:37 PM	AB
Functionality	6	21	15/11/2018 2:40 PM	AB	15/11/2018 3:36 PM	AB
Walking distance	1	2	11/11/2018 8:58 PM	LK	11/11/2018 9:19 PM	LK
Being told- its good-no hospital	1	2	11/11/2018 9:25 PM	LK	11/11/2018 9:26 PM	LK
Maintain within limits	3	3	11/11/2018 9:16 PM	LK	11/11/2018 9:22 PM	LK
Physical appearance	4	6	11/11/2018 8:58 PM	LK	11/11/2018 9:21 PM	LK
Comfort_Min Pain-Able to move	4	8	11/11/2018 9:00 PM	LK	15/11/2018 3:30 PM	AB
Reasons for non compliance	3	5	28/10/2018 5:37 PM	AB	11/11/2018 9:23 PM	LK
Personal reflection of condition	0	0	28/10/2018 5:38 PM	AB	15/11/2018 3:37 PM	AB
People worse off than me	2	4	15/11/2018 3:02 PM	AB	15/11/2018 3:15 PM	AB
Fatalism	4	8	15/11/2018 3:03 PM	AB	15/11/2018 3:32 PM	AB
Normalised condition by using aids that work	1	1	15/11/2018 3:05 PM	AB	15/11/2018 3:24 PM	AB
Changed quality of life	1	1	15/11/2018 3:20 PM	AB	15/11/2018 3:20 PM	AB
Positive state of mind	1	1	15/11/2018 3:29 PM	AB	15/11/2018 3:29 PM	AB
Based on fear level	2	2	15/11/2018 3:32 PM	AB	15/11/2018 3:33 PM	AB
Quality of life	3	8	28/10/2018 5:49 PM	AB	15/11/2018 3:20 PM	AB
Health and medical implications for individual	6	22	28/10/2018 6:03 PM	AB	15/11/2018 3:36 PM	AB
Ulcers-Wounds	2	4	11/11/2018 9:32 PM	LK	8/03/2019 3:29 PM	AB
Falls-Reduced balance	4	18	11/11/2018 9:32 PM	LK	8/03/2019 3:29 PM	AB
Footwear	3	4	11/11/2018 9:40 PM	LK	15/11/2018 2:59 PM	AB
Fear of Falling	1	4	11/11/2018 9:41 PM	LK	11/11/2018 9:42 PM	LK
Cellulitis-Infection	4	7	11/11/2018 9:33 PM	LK	8/03/2019 3:28 PM	AB
Sensitivity-Pain	2	5	11/11/2018 9:34 PM	LK	8/03/2019 3:29 PM	AB
Meaningful outcome	5	8	29/10/2018 11:56 AM	AB	10/11/2018 9:58 PM	LK
